# Crystal structure of a twisted-ribbon type double-stranded Ag^I^ coordination polymer: *catena*-poly[[silver(I)-μ_3_-bis­(pyridin-3-ylmeth­yl)sulfane-κ^3^ *N*:*N*′:*S*] nitrate]

**DOI:** 10.1107/S2056989017013925

**Published:** 2017-09-29

**Authors:** Suk-Hee Moon, Youngjin Kang, Ki-Min Park

**Affiliations:** a Department of Food and Nutrition, Kyungnam College of Information and Technology, Busan 47011, Republic of Korea; b Division of Science Education, Kangwon National University, Chuncheon 24341, Republic of Korea; c Research institute of Natural Science, Gyeongsang National University, Jinju 52828, Republic of Korea

**Keywords:** crystal structure, silver(I), tridentate ligand, double-stranded chain, hydrogen bonding, π–π inter­actions

## Abstract

The asymmetric unit in the title compound, {[Ag(C_12_H_12_N_2_S)]·NO_3_}_*n*_ or {[Ag*L*]·NO_3_}_*n*_, *L* = bis­(pyridin-3-ylmeth­yl)sulfane, consists of an Ag^I^ cation bound to a pyridine N atom of an *L* ligand and an NO_3_^−^ anion that is disordered over two orientations in an 0.570 (17):0.430 (17) occupancy ratio. Each Ag^I^ cation is coordinated by two pyridine N atoms from adjacent *L* ligands to form an infinite zigzag chain along [110]. In addition, each Ag^I^ ion binds to an S donor from a third *L* ligand in an adjacent parallel chain, resulting in the formation of a twisted-ribbon type of double-stranded chain propagating along the [110] or [1-10] directions. The Ag^I^ atom is displaced out of the trigonal N_2_S coordination plane by 0.371 (3) Å because of inter­actions between the Ag^I^ cation and O atoms of the disordered nitrate anions. Inter­molecular π–π stacking inter­actions [centroid-to-centroid distance = 3.824 (3) Å] occur between one pair of corresponding pyridine rings in the double-stranded chain. In the crystal, the double-stranded chains are alternately stacked along the *c* axis with alternate stacks linked by inter­molecular π–π stacking inter­actions [centroid-to-centroid distance = 3.849 (3) Å], generating a three-dimensional supra­molecular architecture. Weak inter­molecular C—H⋯O hydrogen bonds between the polymer chains and the O atoms of the nitrate anions also occur.

## Abstract 2

The reaction of Ag^I^ with the tridentate ligand bis­(pyridin-3-ylmeth­yl)sulfane afforded a one-dimensional double-stranded chain polymeric structure with the charge balanced by a nitrate anion that is disordered over two sites. The Ag^I^ cation adopts a highly distorted trigonal–planar geometry coordinated by two pyridine N atoms and one S atom from three individual ligands. Each ligand bridges the Ag^I^ atoms to form a zigzag chain. Two adjacent chains are connected by an Ag—S bond, resulting in the formation of a twisted-ribbon type double-stranded chain. These chains are linked by π–π inter­actions, generating a three-dimensional supra­molecular network.

## Chemical context   

Among the diverse key factors in the development of Ag^I^ coordination polymers, the structures of the spacer ligands play important roles in determining the structural topology of the self-assembled polymer units (Zheng *et al.*, 2009 ▸; Liu *et al.*, 2011 ▸). For this reason, continuous efforts have focused on the design and development of such suitable ligands. In particular, dipyridyl-type mol­ecules functioning as bridging ligands have been widely used to construct diverse Ag^I^ coordination polymers with fascinating structures and attractive functional properties (Leong & Vittal, 2011 ▸; Moulton & Zaworotko, 2001 ▸; Wang *et al.*, 2012 ▸). We have also reported several Ag^I^ coordination polymers with inter­esting structures using dipyridyl-type ligands (Lee *et al.*, 2012 ▸, 2015 ▸; Moon *et al.*, 2015 ▸, 2016 ▸; Park *et al.*, 2010 ▸). The continuing inter­est in dipyridyl-type-ligand-based Ag^I^ coordination polymers prompted us to investigate the use of the ligand bis­(pyridin-3-ylmeth­yl)sulfane (*L*), which can coordinate to three Ag^I^ cations in a T-shape *via* the two pyridine nitro­gen donors as a bridgehead and the sulfur donor atoms, binding to the Ag^I^ cations at both ends of the dipyridyl bridge as well as at its centre. A reaction of silver(I) nitrate with *L* (synthesized using a literature procedure; Park *et al.*, 2010 ▸; Lee *et al.*, 2012 ▸) afforded the title compound. Herein, we report its one-dimensional twisted-ribbon type double-stranded chain structure in the crystal.
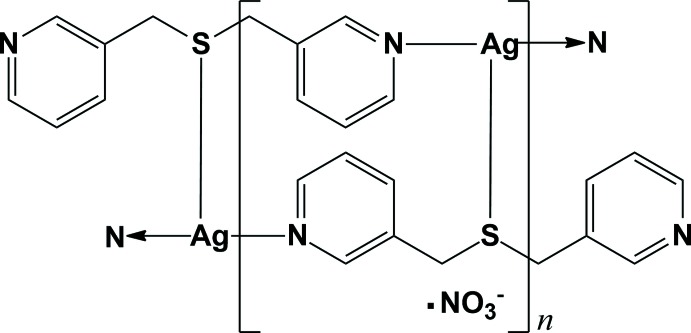


## Structural commentary   

**Figure 1:**
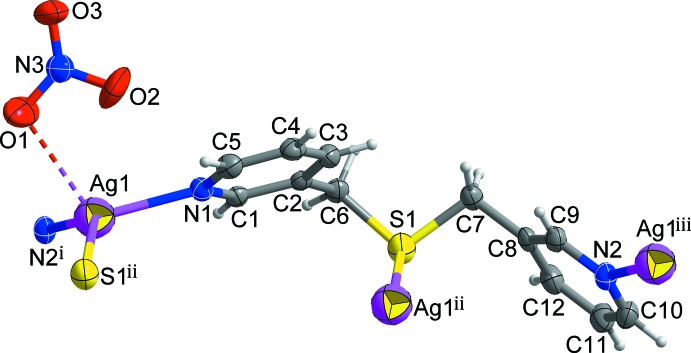
View of the mol­ecular structure of the title compound, showing the atom-numbering scheme. Displacement ellipsoids are drawn at the 50% probability level. Disordered O atoms of the NO_3_^−^ anion have been omitted for clarity. The dashed line represents the Ag⋯O inter­action. [Symmetry codes: (i) *x* − 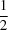, *y* + 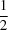, *z*; (ii) −*x* + 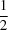, −*y* + 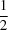, −*z* + 1; (iii) *x* + 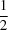, *y* − 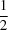, *z*.]

**Figure 2:**
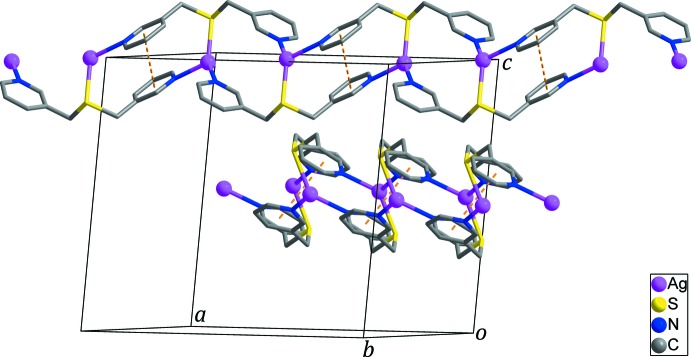
The double-stranded polymeric chains propagating along the [110] (upper chain) and [1
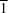
0] (lower chain) directions. Yellow dashed lines represent inter­molecular π–π stacking inter­actions [centroid-to-centroid distance = 3.824 (3) Å] between the N1-containing pyridine rings in the chain. NO_3_^−^ anions and H atoms have been omitted for clarity.

As shown in Fig. 1 ▸, the asymmetric unit of the title compound comprises one Ag^I^ cation, bound to the N1 pyridine atom of a bis­(pyridin-3-ylmeth­yl)sulfane ligand, *L*, and an NO_3_^−^ anion that is disordered over two orientations in an 0.570 (17):0.430 (17) occupancy ratio. Pyridine N atoms N1 and N2 from two symmetry-related *L* ligands bind to the Ag^I^ cations to form an infinite zigzag chain. In addition, each Ag^I^ ion binds to an S1 donor from a third *L* ligand in an adjacent parallel chain, resulting in the formation of a twisted-ribbon type of double-stranded chain propagating along the [110] or [1
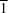
0] directions (Figs. 2 ▸ and 3 ▸). The Ag^I^ atom is therefore three-coordinated and the coordination geometry around the Ag^I^ cation can be considered as a highly distorted trigonal plane. Selected bond lengths and angles around the Ag1 atom are given in Table 1 ▸. N—Ag—N and N—Ag—S angles fall in the range 106.03 (12)–133.18 (12)°, deviating significantly from ideal trigonal–planar geometry. This may reflect the influence of additional Ag⋯O–NO_2_^−^ inter­actions between the Ag^I^ ion and O atoms of the disordered nitrate anion [Ag1⋯O1 = 2.730 (18), Ag1⋯O1′ = 2.55 (2) Å; indicated by a dashed line in Fig. 1 ▸]. The Ag^I^ atom is displaced out of the trigonal N1, S1, N2 coordination plane by 0.372 (2) Å. The two pyridine rings coordinated to the Ag^I^ centre are tilted by 53.20 (15)° with respect to each other. In the double-stranded chain, inter­molecular π–π stacking inter­actions between the N1-pyridine rings [*Cg*1⋯*Cg*1^i^ = 3.824 (3) Å; yellow dashed lines in Fig. 2 ▸; *Cg*1 is the centroid of the N1/C1–C5 ring; symmetry code: (i) −*x* + 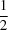, −*y* + 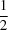, −*z* + 1] contribute to the stabilization of the double-stranded chain.

## Supra­molecular features   

**Figure 3:**
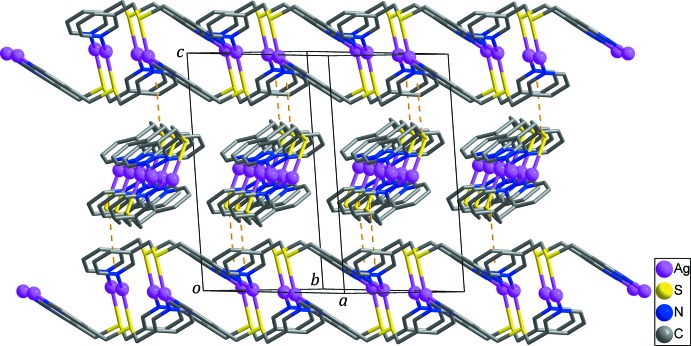
Three-dimensional supra­molecular network *via* inter­molecular π–π stacking inter­actions (yellow dashed lines) between the N2-containing pyridine rings. NO_3_^−^ anions and H atoms have been omitted for clarity.

As shown in Fig. 3 ▸, the double-stranded chains propagate along the [110] and [1
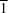
0] directions in the crystal and are alternately stacked along the *c* axis. Adjacent chains are linked by inter­molecular π–π stacking inter­actions between N2-pyridine rings [*Cg*2⋯*Cg*2^ii^ = 3.849 (3) Å; yellow dashed lines in Fig. 3 ▸; *Cg*2 is the centroid of the N2/C8–C12 ring; symmetry code: (ii) −*x* + 1, *y*, −*z* + 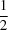], resulting in the formation of a three-dimensional supra­molecular architecture (Fig. 3 ▸). Weak inter­molecular C—H⋯O hydrogen bonds (Table 2 ▸) between the double-stranded chains and the NO_3_^−^ anions are also observed in the crystal.

## Synthesis and crystallization   

The *L* ligand was synthesized according to a literature method (Park *et al.*, 2010 ▸; Lee *et al.*, 2012 ▸). Colourless plate-like X-ray quality single crystals of the title compound were obtained by vapor diffusion of diethyl ether into a DMSO solution of the *L* ligand with AgNO_3_ in a 1:1 molar ratio.

## Refinement   

Crystal data, data collection and structure refinement details are summarized in Table 3 ▸. The NO_3_^−^ anion is disordered over two orientations and the occupancies of the disorder components refined to a 0.570 (17):0.430 (17) ratio. The anisotropic displacement ellipsoids of four oxygen atoms (O3, O1′, O2′ and O3′) in the disordered NO_3_^−^ anion were very elongated and therefore ISOR restraints were applied for these atoms (McArdle, 1995 ▸; Sheldrick, 2008 ▸). All H atoms were positioned geometrically and refined as riding: C—H = 0.93 Å for C*sp* ^2^—H and 0.97 Å for methyl­ene C—H, with *U*_iso_(H) = 1.2*U*_eq_(C).

## Supplementary Material

Crystal structure: contains datablock(s) I, New_Global_Publ_Block. DOI: 10.1107/S2056989017013925/sj5535sup1.cif

Structure factors: contains datablock(s) I. DOI: 10.1107/S2056989017013925/sj5535Isup2.hkl

CCDC reference: 1576726

Additional supporting information:  crystallographic information; 3D view; checkCIF report

## Figures and Tables

**Table 1:** Selected geometric parameters (Å, °)

Ag1—N2^i^	2.276 (5)	Ag1—S1^ii^	2.5305 (14)
Ag1—N1	2.333 (4)		
			
N2^i^—Ag1—N1	113.22 (16)	N1—Ag1—S1^ii^	106.03 (12)
N2^i^—Ag1—S1^ii^	133.18 (12)		

Symmetry codes: (i) 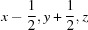; (ii) 
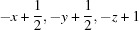
.

**Table 2:** Hydrogen-bond geometry (Å, °)

*D*—H⋯*A*	*D*—H	H⋯*A*	*D*⋯*A*	*D*—H⋯*A*
C5—H5⋯O2	0.93	2.59	2.924 (12)	102
C5—H5⋯O2^iii^	0.93	2.60	3.318 (14)	135
C6—H6*A*⋯O2^iv^	0.97	2.52	3.464 (14)	163
C6—H6*B*⋯O2′^v^	0.97	2.60	3.44 (2)	145
C7—H7*B*⋯O3′^iv^	0.97	2.38	3.233 (15)	147
C9—H9⋯O1^vi^	0.93	2.49	3.221 (18)	136
C12—H12⋯O3^vii^	0.93	2.45	3.256 (12)	145

Symmetry codes: (iii) 
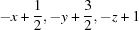
; (iv) 
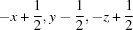
; (v) 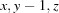; (vi) 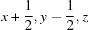; (vii) 
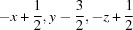
.

**Table 3:** Experimental details

Crystal data	
Chemical formula	[Ag(C_12_H_12_N_2_S)]NO_3_
*M*_r_	386.18
Crystal system, space group	Monoclinic, *C*2/*c*
Temperature (K)	298
*a*, *b*, *c* (Å)	22.432 (3), 8.1656 (12), 15.036 (2)
β (°)	98.636 (3)
*V* (Å^3^)	2722.9 (7)
*Z*	8
Radiation type	Mo *K*α
μ (mm^−1^)	1.64
Crystal size (mm)	0.25 × 0.20 × 0.05

Data collection	
Diffractometer	Bruker APEXII CCD
Absorption correction	Multi-scan (*SADABS*; Bruker, 2014 ▸)
*T*_min_, *T*_max_	0.658, 0.896
No. of measured, independent and observed [*I* > 2σ(*I*)] reflections	7550, 2676, 1763
*R*_int_	0.079
(sin θ/λ)_max_ (Å^−1^)	0.617

Refinement	
*R*[*F* ^2^ > 2σ(*F* ^2^)], *wR*(*F* ^2^), *S*	0.044, 0.109, 0.97
No. of reflections	2676
No. of parameters	209
No. of restraints	24
H-atom treatment	H-atom parameters constrained
Δρ_max_, Δρ_min_ (e Å^−3^)	0.66, −0.66

Computer programs: *APEX2* and *SAINT* (Bruker, 2014 ▸), *SHELXS97* and *SHELXTL* (Sheldrick, 2008 ▸), *SHELXL2014* (Sheldrick, 2015 ▸), *DIAMOND* (Brandenburg, 2010 ▸) and *publCIF* (Westrip, 2010 ▸).

## supplementary crystallographic information

### Crystal data

**Table:**

[Ag(C_12_H_12_N_2_S)]NO_3_	*F*(000) = 1536
*M**_r_* = 386.18	*D*_x_ = 1.884 Mg m^−^^3^
Monoclinic, *C*2/*c*	Mo *K*α radiation, λ = 0.71073 Å
*a* = 22.432 (3) Å	Cell parameters from 3272 reflections
*b* = 8.1656 (12) Å	θ = 1.8–28.3°
*c* = 15.036 (2) Å	µ = 1.64 mm^−^^1^
β = 98.636 (3)°	*T* = 298 K
*V* = 2722.9 (7) Å^3^	Plate, colourless
*Z* = 8	0.25 × 0.20 × 0.05 mm

### Data collection

**Table:**

Bruker APEXII CCD diffractometer	1763 reflections with *I* > 2σ(*I*)
φ and ω scans	*R*_int_ = 0.079
Absorption correction: multi-scan (SADABS; Bruker, 2014)	θ_max_ = 26.0°, θ_min_ = 2.7°
*T*_min_ = 0.658, *T*_max_ = 0.896	*h* = −27→26
7550 measured reflections	*k* = −10→8
2676 independent reflections	*l* = −18→9

### Refinement

**Table:**

Refinement on *F*^2^	24 restraints
Least-squares matrix: full	Hydrogen site location: inferred from neighbouring sites
*R*[*F*^2^ > 2σ(*F*^2^)] = 0.044	H-atom parameters constrained
*wR*(*F*^2^) = 0.109	*w* = 1/[σ^2^(*F*_o_^2^) + (0.0526*P*)^2^] where *P* = (*F*_o_^2^ + 2*F*_c_^2^)/3
*S* = 0.97	(Δ/σ)_max_ < 0.001
2676 reflections	Δρ_max_ = 0.66 e Å^−^^3^
209 parameters	Δρ_min_ = −0.66 e Å^−^^3^

### Special details

**Table:**

Geometry. All esds (except the esd in the dihedral angle between two l.s. planes) are estimated using the full covariance matrix. The cell esds are taken into account individually in the estimation of esds in distances, angles and torsion angles; correlations between esds in cell parameters are only used when they are defined by crystal symmetry. An approximate (isotropic) treatment of cell esds is used for estimating esds involving l.s. planes.

### Fractional atomic coordinates and isotropic or equivalent isotropic displacement parameters (Å^2^)

**Table:**

	*x*	*y*	*z*	*U*_iso_*/*U*_eq_	Occ. (<1)
Ag1	0.13008 (2)	0.47824 (6)	0.49130 (3)	0.05552 (19)	
S1	0.33342 (6)	−0.07112 (16)	0.34926 (9)	0.0417 (3)	
N1	0.2163 (2)	0.4068 (5)	0.4306 (3)	0.0460 (11)	
N2	0.5458 (2)	−0.1552 (6)	0.4256 (3)	0.0501 (11)	
N3	0.1183 (3)	0.8084 (7)	0.3663 (4)	0.0662 (15)	
O1	0.0800 (7)	0.756 (2)	0.4114 (15)	0.106 (6)	0.570 (17)
O2	0.1701 (6)	0.7472 (18)	0.3893 (9)	0.117 (5)	0.570 (17)
O3	0.1169 (6)	0.9256 (15)	0.3181 (8)	0.105 (5)	0.570 (17)
O1'	0.1009 (9)	0.764 (3)	0.4311 (15)	0.072 (5)	0.430 (17)
O2'	0.1641 (12)	0.845 (3)	0.3590 (16)	0.133 (8)	0.430 (17)
O3'	0.0762 (7)	0.815 (2)	0.2930 (9)	0.096 (6)	0.430 (17)
C1	0.2231 (2)	0.2722 (6)	0.3824 (3)	0.0391 (12)	
H1	0.1905	0.2011	0.3699	0.047*	
C2	0.2760 (2)	0.2324 (6)	0.3498 (3)	0.0386 (12)	
C3	0.3244 (2)	0.3392 (6)	0.3697 (4)	0.0464 (13)	
H3	0.3606	0.3175	0.3487	0.056*	
C4	0.3188 (2)	0.4778 (6)	0.4206 (4)	0.0483 (13)	
H4	0.3510	0.5498	0.4349	0.058*	
C5	0.2640 (3)	0.5066 (6)	0.4498 (4)	0.0503 (13)	
H5	0.2601	0.5995	0.4842	0.060*	
C6	0.2786 (2)	0.0785 (7)	0.2966 (4)	0.0466 (13)	
H6A	0.2884	0.1065	0.2378	0.056*	
H6B	0.2389	0.0284	0.2877	0.056*	
C7	0.3975 (2)	−0.0395 (7)	0.2890 (4)	0.0478 (13)	
H7A	0.3870	−0.0722	0.2266	0.057*	
H7B	0.4089	0.0752	0.2908	0.057*	
C8	0.4488 (2)	−0.1419 (6)	0.3345 (3)	0.0426 (12)	
C9	0.4985 (2)	−0.0697 (7)	0.3829 (4)	0.0451 (12)	
H9	0.4997	0.0440	0.3866	0.054*	
C10	0.5422 (3)	−0.3178 (8)	0.4195 (4)	0.0591 (16)	
H10	0.5739	−0.3792	0.4496	0.071*	
C11	0.4946 (3)	−0.4000 (7)	0.3715 (5)	0.0631 (17)	
H11	0.4944	−0.5137	0.3684	0.076*	
C12	0.4474 (3)	−0.3106 (7)	0.3282 (4)	0.0538 (15)	
H12	0.4147	−0.3631	0.2947	0.065*	

### Atomic displacement parameters (Å^2^)

**Table:**

	*U*^11^	*U*^22^	*U*^33^	*U*^12^	*U*^13^	*U*^23^
Ag1	0.0456 (3)	0.0747 (3)	0.0449 (3)	0.0096 (2)	0.00258 (18)	−0.0054 (2)
S1	0.0366 (6)	0.0477 (7)	0.0410 (7)	0.0028 (5)	0.0063 (5)	−0.0036 (6)
N1	0.046 (3)	0.051 (3)	0.042 (3)	0.003 (2)	0.010 (2)	−0.001 (2)
N2	0.043 (3)	0.062 (3)	0.045 (3)	0.007 (2)	0.006 (2)	−0.008 (2)
N3	0.076 (4)	0.060 (4)	0.066 (4)	0.007 (3)	0.020 (4)	−0.003 (3)
O1	0.071 (9)	0.100 (9)	0.147 (15)	−0.021 (7)	0.014 (9)	0.046 (9)
O2	0.102 (8)	0.110 (9)	0.127 (10)	0.063 (7)	−0.016 (7)	0.004 (8)
O3	0.132 (8)	0.088 (7)	0.102 (7)	0.033 (6)	0.045 (6)	0.049 (6)
O1'	0.081 (7)	0.069 (6)	0.068 (6)	−0.005 (5)	0.013 (5)	0.003 (4)
O2'	0.125 (11)	0.145 (11)	0.131 (11)	−0.033 (9)	0.027 (8)	−0.006 (8)
O3'	0.100 (9)	0.110 (9)	0.074 (8)	0.040 (7)	−0.001 (6)	−0.019 (6)
C1	0.037 (3)	0.045 (3)	0.033 (3)	0.003 (2)	0.000 (2)	0.003 (2)
C2	0.041 (3)	0.041 (3)	0.033 (3)	0.006 (2)	0.002 (2)	0.007 (2)
C3	0.042 (3)	0.050 (3)	0.048 (3)	0.001 (2)	0.010 (2)	0.006 (3)
C4	0.046 (3)	0.043 (3)	0.054 (3)	−0.006 (2)	0.003 (3)	0.008 (3)
C5	0.059 (3)	0.047 (3)	0.043 (3)	0.003 (3)	0.004 (3)	−0.004 (3)
C6	0.045 (3)	0.056 (3)	0.037 (3)	0.006 (2)	0.002 (2)	0.000 (3)
C7	0.039 (3)	0.064 (3)	0.040 (3)	0.002 (2)	0.006 (2)	−0.003 (3)
C8	0.037 (3)	0.055 (3)	0.039 (3)	0.001 (2)	0.015 (2)	−0.004 (3)
C9	0.042 (3)	0.051 (3)	0.043 (3)	0.006 (2)	0.009 (2)	−0.005 (3)
C10	0.051 (4)	0.064 (4)	0.063 (4)	0.019 (3)	0.010 (3)	0.005 (3)
C11	0.060 (4)	0.048 (3)	0.085 (5)	0.003 (3)	0.023 (4)	−0.002 (3)
C12	0.045 (3)	0.061 (4)	0.059 (4)	−0.004 (3)	0.018 (3)	−0.012 (3)

### Geometric parameters (Å, º)

**Table:**

Ag1—N2^i^	2.276 (5)	C2—C3	1.389 (7)
Ag1—N1	2.333 (4)	C2—C6	1.495 (7)
Ag1—S1^ii^	2.5305 (14)	C3—C4	1.383 (7)
Ag1—O1'	2.55 (2)	C3—H3	0.9300
S1—C6	1.828 (5)	C4—C5	1.385 (8)
S1—C7	1.829 (5)	C4—H4	0.9300
S1—Ag1^ii^	2.5305 (14)	C5—H5	0.9300
N1—C1	1.338 (6)	C6—H6A	0.9700
N1—C5	1.342 (7)	C6—H6B	0.9700
N2—C10	1.333 (7)	C7—C8	1.500 (7)
N2—C9	1.348 (7)	C7—H7A	0.9700
N2—Ag1^iii^	2.276 (5)	C7—H7B	0.9700
N3—O2'	1.09 (2)	C8—C9	1.371 (7)
N3—O1'	1.16 (2)	C8—C12	1.381 (7)
N3—O3	1.199 (11)	C9—H9	0.9300
N3—O1	1.246 (17)	C10—C11	1.371 (9)
N3—O2	1.265 (12)	C10—H10	0.9300
N3—O3'	1.340 (14)	C11—C12	1.367 (8)
C1—C2	1.389 (7)	C11—H11	0.9300
C1—H1	0.9300	C12—H12	0.9300
			
N2^i^—Ag1—N1	113.22 (16)	C3—C4—H4	120.9
N2^i^—Ag1—S1^ii^	133.18 (12)	C5—C4—H4	120.9
N1—Ag1—S1^ii^	106.03 (12)	N1—C5—C4	123.1 (5)
N2^i^—Ag1—O1'	97.5 (4)	N1—C5—H5	118.4
N1—Ag1—O1'	105.9 (5)	C4—C5—H5	118.4
S1^ii^—Ag1—O1'	95.2 (5)	C2—C6—S1	114.0 (4)
C6—S1—C7	102.7 (3)	C2—C6—H6A	108.7
C6—S1—Ag1^ii^	108.12 (18)	S1—C6—H6A	108.7
C7—S1—Ag1^ii^	105.08 (18)	C2—C6—H6B	108.7
C1—N1—C5	117.6 (5)	S1—C6—H6B	108.7
C1—N1—Ag1	125.9 (3)	H6A—C6—H6B	107.6
C5—N1—Ag1	116.5 (3)	C8—C7—S1	107.6 (4)
C10—N2—C9	116.7 (5)	C8—C7—H7A	110.2
C10—N2—Ag1^iii^	122.9 (4)	S1—C7—H7A	110.2
C9—N2—Ag1^iii^	120.1 (4)	C8—C7—H7B	110.2
O2'—N3—O1'	127.6 (17)	S1—C7—H7B	110.2
O3—N3—O1	130.1 (11)	H7A—C7—H7B	108.5
O3—N3—O2	114.9 (11)	C9—C8—C12	118.2 (5)
O1—N3—O2	113.4 (12)	C9—C8—C7	120.6 (5)
O2'—N3—O3'	117.6 (16)	C12—C8—C7	121.1 (5)
O1'—N3—O3'	114.7 (13)	N2—C9—C8	123.3 (5)
N3—O1'—Ag1	118.8 (14)	N2—C9—H9	118.3
N1—C1—C2	123.8 (5)	C8—C9—H9	118.3
N1—C1—H1	118.1	N2—C10—C11	123.9 (6)
C2—C1—H1	118.1	N2—C10—H10	118.1
C3—C2—C1	117.3 (5)	C11—C10—H10	118.1
C3—C2—C6	123.5 (5)	C12—C11—C10	118.4 (6)
C1—C2—C6	119.2 (5)	C12—C11—H11	120.8
C4—C3—C2	120.0 (5)	C10—C11—H11	120.8
C4—C3—H3	120.0	C11—C12—C8	119.5 (6)
C2—C3—H3	120.0	C11—C12—H12	120.3
C3—C4—C5	118.2 (5)	C8—C12—H12	120.3
			
O2'—N3—O1'—Ag1	−73 (3)	Ag1^ii^—S1—C6—C2	10.9 (4)
O3'—N3—O1'—Ag1	107.3 (14)	C6—S1—C7—C8	173.0 (4)
C5—N1—C1—C2	−1.3 (7)	Ag1^ii^—S1—C7—C8	60.0 (4)
Ag1—N1—C1—C2	−178.0 (4)	S1—C7—C8—C9	−110.0 (5)
N1—C1—C2—C3	0.5 (7)	S1—C7—C8—C12	70.4 (6)
N1—C1—C2—C6	180.0 (5)	C10—N2—C9—C8	−0.5 (8)
C1—C2—C3—C4	0.5 (7)	Ag1^iii^—N2—C9—C8	173.4 (4)
C6—C2—C3—C4	−178.9 (5)	C12—C8—C9—N2	−0.8 (8)
C2—C3—C4—C5	−0.7 (8)	C7—C8—C9—N2	179.6 (5)
C1—N1—C5—C4	1.2 (8)	C9—N2—C10—C11	1.5 (9)
Ag1—N1—C5—C4	178.2 (4)	Ag1^iii^—N2—C10—C11	−172.3 (5)
C3—C4—C5—N1	−0.2 (8)	N2—C10—C11—C12	−1.0 (10)
C3—C2—C6—S1	62.6 (6)	C10—C11—C12—C8	−0.5 (9)
C1—C2—C6—S1	−116.9 (4)	C9—C8—C12—C11	1.3 (8)
C7—S1—C6—C2	−99.9 (4)	C7—C8—C12—C11	−179.1 (5)

Symmetry codes: (i) *x*−1/2, *y*+1/2, *z*; (ii) −*x*+1/2, −*y*+1/2, −*z*+1; (iii) *x*+1/2, *y*−1/2, *z*.

### Hydrogen-bond geometry (Å, º)

**Table:**

*D*—H···*A*	*D*—H	H···*A*	*D*···*A*	*D*—H···*A*
C5—H5···O2	0.93	2.59	2.924 (12)	102
C5—H5···O2^iv^	0.93	2.60	3.318 (14)	135
C6—H6*A*···O2^v^	0.97	2.52	3.464 (14)	163
C6—H6*B*···O2′^vi^	0.97	2.60	3.44 (2)	145
C7—H7*B*···O3′^v^	0.97	2.38	3.233 (15)	147
C9—H9···O1^iii^	0.93	2.49	3.221 (18)	136
C12—H12···O3^vii^	0.93	2.45	3.256 (12)	145

Symmetry codes: (iii) *x*+1/2, *y*−1/2, *z*; (iv) −*x*+1/2, −*y*+3/2, −*z*+1; (v) −*x*+1/2, *y*−1/2, −*z*+1/2; (vi) *x*, *y*−1, *z*; (vii) −*x*+1/2, *y*−3/2, −*z*+1/2.
